# Norovirus Diversity in Diarrheic Children from an African-Descendant Settlement in Belém, Northern Brazil

**DOI:** 10.1371/journal.pone.0056608

**Published:** 2013-02-15

**Authors:** Glicélia Cruz Aragão, Joana D'Arc Pereira Mascarenhas, Jane Haruko Lima Kaiano, Maria Silvia Sousa de Lucena, Jones Anderson Monteiro Siqueira, Túlio Machado Fumian, Juliana das Mercês Hernandez, Consuelo Silva de Oliveira, Darleise de Souza Oliveira, Eliete da Cunha Araújo, Luana da Silva Soares, Alexandre Costa Linhares, Yvone Benchimol Gabbay

**Affiliations:** 1 Universidade do Estado do Pará, Belém, Pará, Brazil; 2 Instituto Evandro Chagas, Ananindeua, Pará, Brazil; 3 Núcleo de Medicina Tropical/UFPA, Belém, Pará, Brazil; 4 Instituto de Ciências da Saúde/UFPA, Belém, Pará, Brazil; University of Malaya, Malaysia

## Abstract

Norovirus (NoV), sapovirus (SaV) and human astrovirus (HAstV) are viral pathogens that are associated with outbreaks and sporadic cases of gastroenteritis. However, little is known about the occurrence of these pathogens in relatively isolated communities, such as the remnants of African-descendant villages (“Quilombola”). The objective of this study was the frequency determination of these viruses in children under 10 years, with and without gastroenteritis, from a “Quilombola” Community, Northern Brazil. A total of 159 stool samples were obtained from April/2008 to July/2010 and tested by an enzyme immunoassay (EIA) and reverse transcription-polymerase chain reaction (RT-PCR) to detect NoV, SaV and HAstV, and further molecular characterization was performed. These viruses were detected only in the diarrheic group. NoV was the most frequent viral agent detected (19.7%-16/81), followed by SaV (2.5%-2/81) and HAstV (1.2%-1/81). Of the 16 NoV-positive samples, 14 were sequenced with primers targeting the B region of the polymerase (ORF1) and the D region of the capsid (ORF2). The results showed a broad genetic diversity of NoV, with 12 strains being classified as GII-4 (5–41.7%), GII-6 (3–25%), GII-7 (2–16.7%), GII-17 (1–8.3%) and GI-2 (1–8.3%), as based on the polymerase region; 12 samples were classified, based on the capsid region, as GII-4 (6–50%, being 3–2006b variant and 3–2010 variant), GII-6 (3–25%), GII-17 (2–16.7%) and GII-20 (1–8.3%). One NoV-strain showed dual genotype specificity, based on the polymerase and capsid region (GII-7/GII-20). This study provides, for the first time, epidemiological and molecular information on the circulation of NoV, SaV and HAstV in African-descendant communities in Northern Brazil and identifies NoV genotypes that were different from those detected previously in studies conducted in the urban area of Belém. It remains to be determined why a broader NoV diversity was observed in such a semi-isolated community.

## Introduction

Acute gastroenteritis (AGE) is a common childhood disease that causes high rates of hospitalization and mortality, particularly in less-developed regions of the world. Each year, AGE is responsible for about 1.4 million deaths, occurring primarily in low and middle income countries [1], [2]. Although different groups of pathogens are involved in the etiology of AGE, enteric viruses play a key role as causative agents. The most relevant of these enteric viruses are group A rotavirus (RV-A), norovirus (NoV), adenovirus types 40/41 (AdV-40/41), human astrovirus (HAstV) and sapovirus (SaV) [3]. In settings where most of the population lives under conditions of poor hygiene, including poor quality water, food and sanitation, these viruses are common causes of AGE, which occurs most often in the first year of life [4], [5].

NoV and SaV, also known as human caliciviruses (HuCV), are classified in the *Caliciviridae* family. These viruses are responsible for causing outbreaks and sporadic cases of acute viral gastroenteritis in humans. NoV is recognized as the major cause of extensive AGE outbreaks that involve different settings, such as restaurants, nursing homes, schools and cruise ships [6], [7], [8], [9]. Recent studies have emphasized the role of NoV as a cause of AGE in hospitalized children [10], [11], [12], [13]. NoV strains are classified into five genogroups (GI-GV) on the basis of sequence similarity, of which the GI, GII and GIV genogroups are associated with human infections [14]. These genogroups are further divided into at least 32 genotypes; however, genotype GII-4 has emerged as the dominant strain worldwide, responsible for 70–80% of NoV outbreaks during the past 20 years [15], [16], [17], [18].

Enteric viruses are transmitted by the fecal-oral route, primarily through contaminated food and water, as well as through person-to-person spread [9]. Contaminated food is a high-risk source for the occurrence of outbreaks, especially those caused by NoV [19], [20].

Studies on the molecular epidemiology of enteric viral infections in hospitals, outpatient health units and the community have already been conducted [3], [5], [7]; nevertheless, studies on the epidemiology of infection in semi-closed communities such as Indian villages and African-descendant settlements are lacking.

The African-descendant settlements, known as “Quilombola” communities in Brazil, are rural communities that are composed of the descendants of African slaves. Members of these communities primarily survive through subsistence agricultural activity, with cultural traditions that preserve a strong link with the past. These social groups show ethnic and cultural identities that are distinct from the rest of the society [21].

This study aimed to assess the frequency of NoV, SaV and HAstV in cases of AGE among children living in a “Quilombola” community in the State of Pará, Brazil. This is the first study in the Amazon region, Northern Brazil that aims to detect enteric viruses in African-descendant communities.

## Patients, Materials and Methods

### Study population

**Figure 1:**
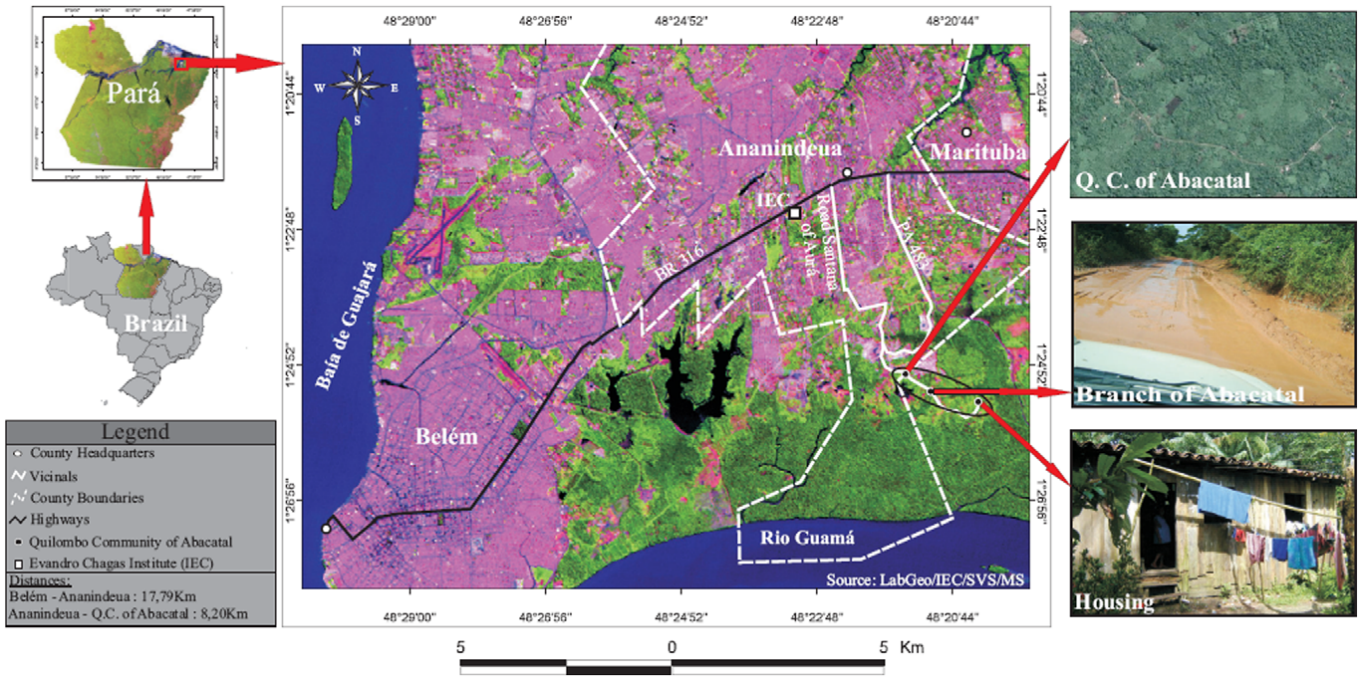
Map showing the location of the Abacatal “Quilombola” Community, in the metropolitan region of Belém city, Pará State, Amazon region, Brazil.

This study was conducted in the semi-closed Abacatal “Quilombola” Community, located in the outskirts of Ananindeua and Marituba municipalities, which belong to the metropolitan area of Belém, Pará State, Amazon region, Brazil. This community is located 8.2 km from downtown Ananindeua (Figure 1).

The community occupies an area of 308 hectares (01°25′18″S e 48°20′58″W) and includes 84 families with a population of about 400 persons, including about 120 children aged less than 10 years. An official stratified data involving this subgroup is not available, but those individuals from whom fecal samples (n = 159) were obtained, were distributed as follows: 0–1 (14.5%), >1–5 (55.3%) and >5–10 (30.2%) years.

The socioeconomic level is very low, and the economy is based on agricultural subsistence activity (vegetables and fruits). These products are commercialized once a week in a free market in the Ananindeua municipality. Most of the families live in wooden or clay houses and have close contact with domestic animals, such as dogs, cats, chickens, ducks and pigs. Sewage infrastructure is not available, and access to potable water is lacking. Medical assistance is provided by health units located in downtown Ananindeua. Accessing this community is difficult, as it is accessible only through one land road that is difficult to access during the rainy season. The “Quilombola” have contact with outside urban communities during the sale of their products and through young people who attend high school in Ananindeua.

### Patients and clinical specimens

This study was conducted from April/2008 to July/2010. Fecal samples were obtained during twice-a-week visits to the village by two pediatricians, who provided treatment to children with diarrhea. Samples were also obtained during active surveillance for diarrheic cases (three or more liquid or semi-liquid evacuations in a 24 hours period), also carried out twice-a-week in the community. For each diarrheic case collected, one normal fecal specimen (asymptomatic case) from an individual of similar age was obtained to be used as a control. The samples were refrigerated in iceboxes and brought to the Virology Section, Evandro Chagas Institute, and kept frozen at −20°C until virus analysis.

### Ethics

The Ethics Committee on Human Research of the Evandro Chagas Institute (IEC-CEPH) granted ethical approval to our study under number 0024.0.072.000-06. The ethical consent form was applied to the subjects of this research. Initially, the study team held meetings with community members, such as community leaders, health visitors and school directors, in order to obtain a better understanding of the study area and to inform them about the research objectives. Written informed consent was signed by parents or guardians of the children during the fecal specimen collection.

### NoV antigen detection

The detection of NoV was initially performed using a commercial enzyme immunoassay (EIA) (Ridascreen®Norovirus 3^rd^ Generation, R-Biopharm AG, Darmstadt Germany), following the manufacturer's instructions. The kit is a sandwich EIA whereby specific monoclonal antibodies against NoV antigens GI and GII came adhered to the micro-wells. Briefly, 10% (wt/vol) fecal suspensions were prepared with the diluent solution (NaCl buffer) and added into the wells together with an enzyme-labeled polyclonal antibody. Following incubation, a horseradish peroxidase conjugate was used for detection. The results were visually read and confirmed by absorbance measurements [22].

### Viral RNA extraction and reverse transcription (RT)

Viral RNA was extracted from a 10% fecal suspension (the same as used in the EIA test) using a guanidine isothiocyanate/silica method [23], followed by cDNA synthesis performed using a pd(N)_6_™ random primer (Amersham Biosciences, UK) and the Superscript™ II RNAse H Reverse (Invitrogen, USA).

### Molecular detection and characterization

The viral genome was detected by polymerase chain reaction (PCR) using the following primers: p289/p290 to detect HuCV (NoV and SaV) [24]; Mon 431–434 to detect NoV GI and GII [25]; and Mon 269/270 to detect HAstV [26]. PCR was carried out using the Taq DNA polymerase (Biotools, Spain). NoV-positive samples by EIA or PCR were also tested by another PCR using a set of primers that targeted the capsid region [27].

The PCR amplicons obtained from PCR-positive samples were purified using the commercial kits QIAquick® Gel Extraction Kit or QIAquick® PCR Purification Kit (QIAGEN, CA, USA), as recommended by the manufacturers. The purified DNA was subjected to a sequencing reaction, in both directions, using a Big Dye Terminator Cycle Sequencing Ready Reaction Kit® (v.3.1) (Applied Biosystems) and an ABI Prism 3130 Genetic Analyzer (Applied Biosystems, Foster City, USA). The chromatograms were analyzed, and sequences were edited using the BioEdit Sequence Alignment Editor (v.7.0.9.1) software. A dendogram was constructed by the neighbor-joining method using a matrix of genetic distances established under the Kimura-two parameter model using MEGA 5.05 [28], [29]. The robustness of each node was assessed by bootstrap analysis using 2,000 pseudo-replicates. All sequenced strains were also compared with sequences available in the GenBank database, including reference strains. A nucleotide sequence Basic Local Alignment Tool (BLAST) search was performed for each NoV strain detected, and strains were also analyzed using the automated typing-tool available on line (http://www.rivm.nl/mpf/norovirus/typingtool), which is recommended for genotyping nomenclature harmonization [30].

The nucleotide sequences obtained in this study were deposited into the National Center for Biotechnology Information (GenBank: http://www.ncbi.nlm.nih.gov/) under the accession number JX047011-JX047023.

### Statistical analysis

Statistical analyses were performed using the software BioEstat 5.0 [31]. The G test (G) was applied to analysis correlating the ages of children to NoV detection. Odds Ratio (OR) was used to compare the prevalence between the Brazilian school-vacation months (January, February and July) and the other months of the year; p-values≤0.05 were considered to be statistically significant.

## Results

Of the 159 samples collected, 81 and 78 came from 50 diarrheic and 57 non-diarrheic children, respectively. In the diarrheic group, one, two, three, and four stool samples were collected from 30, 12, 5, and 3 children, respectively. All samples from non-diarrheic children were negative for all enteric viruses tested. NoV, SaV and HAstV were detected in 23.5% (19/81) of fecal samples obtained from diarrheic children. NoV was the most frequently detected pathogen (19.7%-16/81), followed by SaV (2.5%-2/81) and HAstV (1.2%-1/81).

Of the samples that were positive for NoV, eight (50%) were detected by both EIA and PCR (with primers specific for the polymerase and capsid region); seven (43.7%), only by PCR; and one (6.3%), by EIA only (Table 1).

**Table 1:** Detection of norovirus in fecal specimens from diarrheic children from the Abacatal “Quilombola” Community, according to the methodology used. April/2008 to July/2010.

Date collection Month/day/year	Sample register	Detection			Partial sequencing	
		EIA	PCR(B region)	PCR(D region)	Sequenced region	Genotypes
07/30/2008	QUI 23 F2	−	+	−	B	GII-7
08/08/2008	QUI 27 F1	−	+	−	—	NT^a^
08/08/2008	QUI 29 F1	−	+	+	B+D	GII-6/GII-6
08/11/2008	QUI 13 F3	+	+	+	B+D	GII-6/GII-6
08/11/2008	QUI 14 F2	−	+	+	B+D	GII-6/GII-6
08/11/2008	QUI 38 F1	+	+	+	B+D	GII-7/GII-20
11/06/2008	QUI 56 F2	+	+	+	B+D	GII-4/GII-4 2010
01/15/2009	QUI 72 F1	−	+	−	B	GI-2
01/22/2009	QUI 13 F4	−	+	+	D	GII-17
01/22/2009	QUI 78 F1	−	+	+	B+D	GII-17/GII-17
08/27/2009	QUI 108 F1	+	−	−	—	NT^a^
01/21/2010	QUI 26 F5	+	+	+	B+D	GII-4/GII-4 2006b
01/21/2010	QUI 65 F3	+	+	+	B+D	GII-4/GII-4 2006b
01/21/2010	QUI 106 F4	+	+	+	B+D	GII-4/GII-4 2006b
02/11/2010	QUI 126 F1	+	−	+	D	GII-4 2010
02/18/2010	QUI 71 F5	+	+	+	B+D	GII-4/GII-4 2010

a NT- not typed.

**Figure 2:**
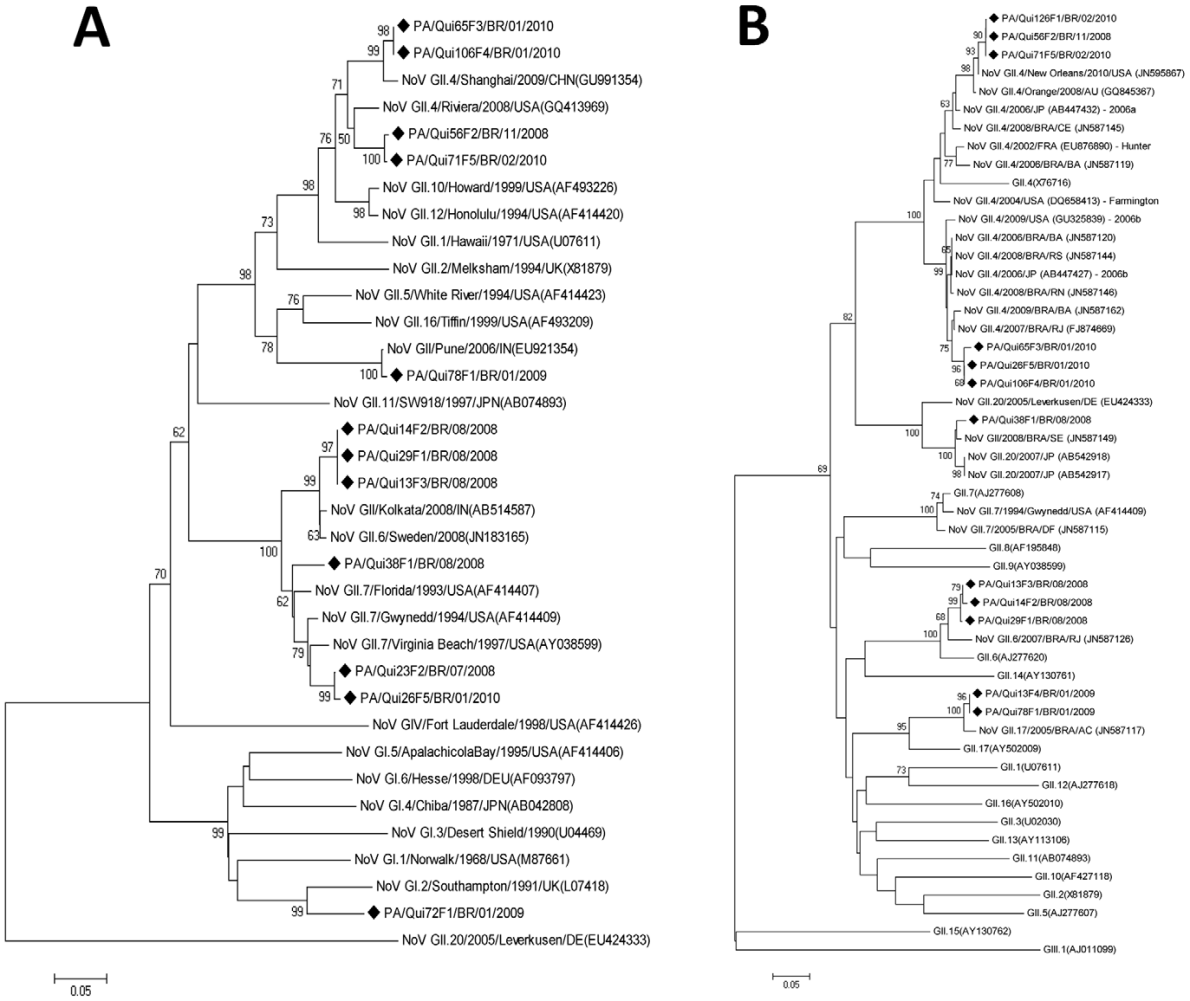
Dendograms constructed using the partial region of the norovirus RNA polymerase sequence (ORF1-B) (A) and capsid region (ORF2-D) (B) amplified from 14 strains from diarrheic children of Abacatal “Quilombola” Community, metropolitan region of Belém, Pará State, Brazil. April/2008 to July/2010. Prototype strains are presented together with strains from other locations. The number above each branch corresponds to the bootstrap value (2,000 replicates). The scale bar is proportional to the genetic distance. Study samples were marked (♦): PA-local/Qui-code in the study+F_x_-Number of the stool/country/month+year of collection.

NoV genotype characterization showed a broad genetic diversity and a significant circulation of genotypes other than the GII-4. Partial nucleotide sequencing of the polymerase gene using the primers Mon 431–433 classified 12 strains as GII-4 (5–41.7%), GII-6 (3–25%), GII-7 (2–16.7%), and GII-17 (1–8.3%) and one strain as GI-2 (1–8.3%). Using primers for the capsid region (partial VP1 gene), 12 samples were genetically characterized as follows: six (50%) as GII-4 [variants 2006b (n = 3) and 2010 (n = 3)], three (25%) as GII-6, two (16.7%) as GII-17, and one (8.3%) as GII-20. Ten of these positive samples were characterized by both polymerase and capsid genes (ORF1/ORF2 partial nucleotide sequencing). Nine strains were classified as the same genotype by both analyzed regions; however, one strain grouped in a different genotype: GII-7/GII-20 (Figure 2a and 2b). The two SaV-positive samples were sequenced and classified as genotypes GI-1 and GII-2, and the HAstV strain as genotype 3.

NoV was detected primarily in fecal samples from children in the 0–1 year (23.5%) and >1–5 years (20%) age groups, while SaV and HAstV were detected in the >1–5 years age group. No statistically significant difference was observed when comparing the NoV frequencies among each age group (G = 0.0094; p = 0.9228) (Table 2).

**Table 2:** Distribution by age group of positive samples for norovirus, sapovirus and astrovirus detected in children from the Abacatal “Quilombola” Community.

Age/years	NoV	SaV	HAstV
	Pos/tested (%)	Pos/tested (%)	Pos/tested (%)
0–1	4/17 (23.5)	0/17	0/17
>1–5	10/50 (20)	2/50 (4)	1/50 (2)
>5–10	2/14 (14.3)	0/14	0/14
Total	16/81	2/81	1/81

Pará State, Brazil, April/2008 to July/2010.

**Figure 3:**
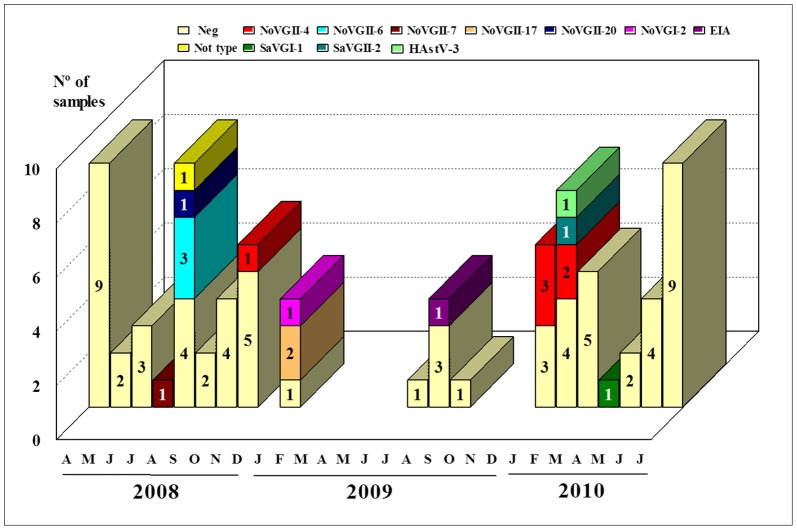
Monthly distribution of the positive cases of norovirus, sapovirus and astrovirus (including their genotypes) detected in stool specimens from diarrheic children of the Abacatal “Quilombola” Community, Pará State, Brazil. April/2008 to July/2010.

The monthly distribution of NoV-positive samples denoted a higher prevalence in the months of August/2008, January/2009 and January-February/2010; SaV was detected in the months of February and April/2010, and HAstV was detected in February/2010 (Figure 3). A statistically significant difference was observed when the positivity for NoV in the school-vacation months (January, February and July) was compared with the positivity in other months of the year (OR = 11.86; p<0.0004).

It was observed that samples classified as NoV GII-6 were all detected in August/2008 and GII-17 in January/2009. Most of the GII-4 strains circulate in January and February/2010.

## Discussion

Our study showed that NoV circulates at high rates and found a broad genetic diversity among children with gastroenteritis disease in a semi-closed “Quilombola” community. NoV, SaV and HAstV were only detected in diarrheic individuals, with a positivity of 19.7%, 2.5% and 1.2%, respectively. Rotavirus showed a positivity of 4.7% [32], lower than the rates observed for NoV. Epidemiological studies involving the detection of viral gastroenteritis pathogens in the “Quilombola” population are still rare, as most of the studies have been conducted in hospitals and health units in urban settings.

The results obtained in this study for NoV (19.7%) were higher than the partial results demonstrated in another study that is still under way in different “Quilombola” communities in Espírito Santo State, Southeastern Brazil. In these communities, NoV was detected in both symptomatic (8.5%) and asymptomatic groups (2.3%) [33]. A study conducted in Belém involving hospitalized and non-hospitalized children with AGE in 2003 demonstrated a NoV detection rate of 12.5%, lower than the results obtained in the Abacatal community [10]. In a seroepidemiological survey involving eight isolated Indian tribes in the Amazon region, six located in Pará State, one in Amazonas State and one in Venezuela, the NoV positivity ranged from 39% to 100% [34].

The broad diversity of NoV genotypes found in the present study is in contrast with results obtained previously in other studies conducted in Belém, where GII-4 was the dominant circulating genotype, aside from a low circulation of other genotypes [10], [22]. On the other hand, our results agree with those from previous recent studies where NoV GII-4, albeit predominant, co-circulates with other genotypes [35], [36]. However, most of these studies included either hospitalized children or outbreaks of AGE [10], [37], [38], [39].

The genetic diversity of NoV was recently described in two studies: one that tested fecal samples from several Brazilian states and one that examined specimens from Rio de Janeiro State [40], [41]. Those authors observed a predominance of GII-4 (78% and 80.7%, respectively), which co-circulated with other genotypes. Some of these genotypes (GII-6, GII-7, and GII-17) were also detected in the Abacatal community, in addition to genotype GII-20. One NoV strain (QUI 38 F1) showed different genotype specificities for its polymerase and capsid regions (GII-7/GII-20), revealing a recombination event by partial sequencing of the ORF1/2 junction region and SimPlot analysis [42].

The GII-4 variants (2006b and 2010) detected in this study have a worldwide distribution and are mainly related to outbreaks and epidemics of AGE [43], [44]. The GII-4 2010 variant showed a 99% similarity with the nucleotide sequences of samples described in 2009, such as GII-4 New Orleans, which is also associated with AGE outbreaks [45].

One child developed two NoV infections: one by genotype GII-17, in August/2008, and a subsequent infection by GII-6, in January/2009. This finding highlights the concept that naturally induced cross-protection does not occur in NoV infections. Interestingly, both genotypes had been previously detected in Brazil in the states of Acre and Rio de Janeiro, in 2005 and 2007, respectively [40], [46]. The genotype GII-6 has also been detected in studies conducted in China (2008–2009), Japan and India (2007–2009) [47], [48], [49].

One isolate of NoV GI-2 was also found. This genotype is detected less often compared to NoV GII. In a surveillance study conducted in Belém city [10], only one sample was found to be GI-4, while in Rio de Janeiro, two GI-2 samples were among hospitalized children during a period of four years (2005 to 2008) [37].

Most of the NoV positive cases were detected during the Brazilian school vacation months (January, February and July) or just thereafter (August). It is likely that, in this period, children and several families resident in the “Quilombola” community are more likely to travel outside the village, providing more exposure to infection and a greater possibility for introducing NoV to the community. The OR value demonstrated that the probability of NoV infection in the community is highest in the vacation months, with individuals almost 12 times more likely to be affected by the disease. However, during 2009, fecal specimens were not collected in certain months as a result of heavy rainfall, which posed some logistical problems for gaining access to the community. The lack of complete information prevented us from properly assessing seasonality. The fact that some diarrheic cases were missed also represents a limitation of the study.

The search for SaV is still limited in Brazil, with few studies conducted to date. In the “Quilombola” community, SaV accounted for 2.5% of the pathogens detected in the diarrheic cases. This result is lower than those previously observed in Belém (3%, 4.9%, and 8.9%) and higher than rates observed in Salvador (0.7%) [10], [50], [51], [52].

The two cases that were positive for SaV were detected in the same child during diarrheic episodes, in two separate months (February and April) and with two different genotypes (GI-1 and GII-2). Thus, the first infection may not have elicited immunity that was sufficient to prevent reinfection.

A single sample was HAstV-3-positive. This genotype has been detected only rarely in Belém: in a study conducted for 18 years in Belém, this type was detected in 4.2% of the positive samples, while genotype 1 was detected in 60.6% of cases [53].

Our study provided evidence of the co-circulation of major enteric viral pathogens in a semi-closed African-descendant community, underscoring the need for a continuous surveillance, especially considering the relatively recent introduction of rotavirus vaccination in Brazil. The NoV-related findings (high rate, broad diversity, reinfection) are relevant and, as a consequence, a challenge for current efforts toward the development of a vaccine.
